# Acute kidney injury and length of ICU stay in diabetic ketoacidosis: a retrospective cohort study

**DOI:** 10.3389/fendo.2026.1872959

**Published:** 2026-08-04

**Authors:** Nosayba Al-Azzam, Sayer Al-Azzam, Reema Karasneh, Malek AlNajjar, Mallak Alzubi, Aren Abu Sarieh, Yamin Al-Azzam

**Affiliations:** 1 Department of Physiology and Biochemistry, Faculty of Medicine, Jordan University of Science and Technology, Irbid, Jordan; 2 Department of Clinical Pharmacy, Faculty of Pharmacy, Jordan University of Science and Technology, Irbid, Jordan; 3 Department of Basic Pathological Sciences, Faculty of Medicine, Yarmouk University, Irbid, Jordan; 4 Faculty of Medicine, Jordan University of Science and Technology, Irbid, Jordan

**Keywords:** diabetic ketoacidosis, acute kidney injury, intensive care unit, length of stay, hyperglycemia

## Abstract

**Aims:**

Diabetic ketoacidosis (DKA) is a major acute complication of diabetes mellitus and a leading cause of diabetes-related morbidity. Acute kidney injury (AKI) further compounds this burden, yet data on AKI and prolonged intensive care unit (ICU) stay in DKA from the Middle East remain scarce. We aimed to evaluate AKI prevalence and identify predictors of AKI and prolonged ICU stay among adults admitted to the ICU with DKA at a Jordanian tertiary hospital.

**Methods:**

This retrospective cohort study included 183 adults admitted to the ICU with DKA (2012–2024) at King Abdullah University Hospital, Jordan. AKI was defined by Kidney Disease: Improving Global Outcomes (KDIGO) creatinine criteria; prolonged ICU stay was defined as a total ICU length of stay of ≥3 days. Multivariable logistic regression identified independent predictors.

**Results:**

AKI occurred in 15.9% of patients and 28.7% had prolonged ICU stay. Ninety-day mortality was 4.4%. Elevated admission glucose independently predicted AKI [adjusted odds ratio (aOR) 1.05 per mmol/L; 95% confidence interval (CI) 1.00–1.10; *p* = 0.034]. Predictors of prolonged ICU stay were AKI (aOR 4.88; 95% CI 1.73–13.77; *p* = 0.003), infection (aOR 4.00; 95% CI 1.83–8.76; *p* < 0.001), elevated sodium (aOR 1.07; 95% CI 1.00–1.14; *p* = 0.039), and lower potassium (aOR 0.52, 95% CI 0.28–0.97, *p* = 0.040). Higher sodium levels, AKI incidence, and infection were robust across the ≥2-day threshold, infection was robust also at the ≥4-day threshold, while potassium was significant only at the ≥3-day definition.

**Conclusions:**

Among ICU-admitted patients with DKA, AKI affected one in six and was a strong independent predictor of extended hospitalization. Admission hyperglycemia, infection, and hypernatremia are accessible markers that may support early risk stratification, though these exploratory associations warrant prospective validation.

## 1 Introduction

Diabetic ketoacidosis (DKA) is the most common acute hyperglycemic emergency in people with diabetes mellitus and remains an important contributor to diabetes-related morbidity, healthcare utilization, and mortality. It is characterized by hyperglycemia, metabolic acidosis, and ketonemia resulting from an absolute or relative lack of insulin (1). According to the American Diabetes Association (ADA), DKA remains a common cause of hospital admissions among patients with both type 1 and type 2 diabetes (2) and continues to contribute substantially to healthcare utilization and acute morbidity worldwide (3).

DKA is classified as mild, moderate, or severe based on the degree of acidosis (serum pH and bicarbonate) and altered mental status (4). While mild to moderate presentations can often be managed in general medical settings, severe DKA frequently may necessitate admission to the intensive care unit (ICU) due to profound metabolic and electrolyte derangements, hemodynamic instability, and impaired mental activity (5). Additional determinants of ICU-level care include age, new-onset diabetes, diabetes type, and acute triggers such as infection (6).

Acute kidney injury (AKI) is one of the most common and clinically significant complications of DKA (7). Volume depletion resulting from osmotic diuresis, hypotension, electrolyte disturbances, and underlying comorbidities may predispose these patients to renal dysfunction (7, 8). The reported incidence of AKI in patients with DKA varies across studies. Across contemporary studies using creatinine-based definitions, AKI occurs in roughly one-third to one-half of general hospitalized DKA episodes, and approximately half or higher of severe or ICU-treated DKA (7, 9–12). Renal impairment has been consistently linked to increased morbidity, longer hospital stays, and worse clinical outcomes (9). Early identification of patients at risk for AKI is therefore essential to guide fluid management and optimize supportive care (13).

Prolonged ICU stay is an independent contributor to morbidity, systemic complications, higher healthcare expenditure, and increased resource utilization (14). Several factors, including severity of acidosis, infection, and renal dysfunction, have been associated with extended hospitalization in patients with DKA (15, 16).

Although the global burden of DKA is well described, most available data regarding renal complications and ICU resource utilization originate from Western populations. Data from the Middle East remain limited. Therefore, this study aimed to evaluate the prevalence of AKI and to identify independent predictors of AKI and length of stay in ICU-admitted patients with DKA at King Abdullah University Hospital (KAUH), Irbid, Jordan.

## 2 Methods

### 2.1 Study design and setting

This is a retrospective observational cohort study conducted in the adult ICU of KAUH, a tertiary teaching hospital in Irbid, Jordan. The study was approved by the Institutional Review Board of Jordan University of Science and Technology (2026/190/46). Given the retrospective nature of the study, the requirement for informed consent was waived. All data were de-identified prior to analysis to ensure patient confidentiality, and the study adhered to the ethical principles outlined in the Declaration of Helsinki.

### 2.2 Patient selection

Patients admitted to the ICU from 1 July 2012 to 31 December 2024 who were aged 18 years or older and had a primary diagnosis of DKA, as coded by ICD-10 criteria, were included in this study. Patients with gestational diabetes were excluded. Only first ICU admissions during the study period were included.

### 2.3 Data collection

Patient data were extracted from the hospital’s electronic medical records (EMRs) and supplemented by manual review of physician and discharge notes. Collected variables included demographic data (age, sex, and body mass index when available), diabetes characteristics (type and duration), comorbidities [hypertension, chronic kidney disease (CKD), and ischemic heart disease (IHD)], DKA precipitating factors (infection, insulin non-adherence, and new-onset diabetes), clinical status at ICU admission [vital signs, Glasgow Coma Scale (GCS), vasopressor use, and invasive mechanical ventilation], severity scores (Sequential Organ Failure Assessment (SOFA) and Acute Physiology and Chronic Health Evaluation (APACHE) II), and the earliest value recorded of laboratory variables during ICU admission (glucose, arterial/venous blood gas parameters including pH, and serum sodium, potassium, and creatinine). HbA1c readings recorded within a 3-month window around the index admission were extracted from patient records. Infection as a precipitating factor was defined on the basis of the treating physician’s documented clinical diagnosis in the medical record, supported where available by laboratory, radiological, or microbiological findings, rather than requiring microbiological confirmation. Isolated positive cultures without a corresponding documented clinical diagnosis were not classified as a precipitating infection.

### 2.4 Outcome definitions

Primary outcomes were the occurrence of AKI during the ICU admission and prolonged ICU stay. AKI was defined in accordance with the Kidney Disease: Improving Global Outcomes (KDIGO) 2012 criteria using serial serum creatinine measurements. AKI was identified as an absolute increase in serum creatinine of ≥26.5 µmol/L (≥0.3 mg/dL) within 48 h. The admission serum creatinine served as the baseline value in all cases. As urine output data were not consistently documented in the EMRs, AKI staging was based exclusively on serum creatinine criteria; this is acknowledged as a limitation. Baseline renal function was identified using initial measurement documented upon admission. Because of the retrospective design, a stable pre-admission or outpatient (baseline) serum creatinine was not reliably available for the majority of patients; the first creatinine measured at ICU admission was therefore used as the baseline reference. We acknowledge that, in patients presenting with marked volume depletion, this approach may classify some community-acquired AKI as baseline renal function and may not capture unrecognized pre-existing CKD. Both would tend to underestimate, rather than inflate, the AKI incidence reported here. Documented CKD was present in 7 patients (3.8%), and the admission creatinine exceeded 106 µmol/L (≈1.2 mg/dL) in 41 of 181 patients (22.7%), indicating that overt renal dysfunction at presentation was present in a substantial minority of patients in this cohort.

Prolonged ICU stay was defined as a total ICU length of stay of ≥3 days. This threshold was pre-specified relative to the observed cohort median ICU stay of 2.0 days, so that the outcome identified patients whose stay exceeded the duration expected for uncomplicated DKA, which typically resolves within 24–72 h of guideline-based therapy (17, 18). To confirm that the findings were not dependent on this particular cutoff, we performed sensitivity analyses repeating the multivariable model across alternative thresholds (≥2, ≥4, and ≥5 days) (Section 3.5).

Secondary outcomes included 90-day all-cause mortality and 90-day hospital readmission. Readmission and mortality were analyzed descriptively only; because of the small number of events (13 readmissions and 8 deaths), no multivariable models were fitted for these outcomes, and the reported association between AKI and 90-day readmission should be regarded as exploratory and hypothesis-generating.

### 2.5 Severity of illness assessment

Severity of illness was assessed at ICU admission using two validated scoring systems: a modified SOFA score and a modified APACHE II score. Both scores were calculated from clinical and laboratory data documented at the time of ICU admission.

#### 2.5.1 SOFA score

The modified SOFA score was calculated across six organ systems, each scored from 0 to 4 points based on the degree of dysfunction, yielding a total score ranging from 0 to 24, with higher scores reflecting greater organ failure burden (19). Respiratory function was scored using the PaO_2_/FiO_2_ ratio (≥400 = 0; 300–399 = 1; 200–299 = 2; 100–199 = 3; <100 = 4). Coagulation was assessed by the lowest platelet count in ×10^9^/L (≥150 = 0; 100–149 = 1; 50–99 = 2; 20–49 = 3; <20 = 4). Hepatic function was evaluated using the highest total serum bilirubin in µmol/L (<20.5 = 0; 20.5–32.5 = 1; 33–101 = 2; 102–204 = 3; >204 = 4). Cardiovascular function was assessed using mean arterial pressure (MAP), with a score of 0 assigned for MAP ≥70 mmHg and 1 for MAP <70 mmHg. Vasopressor use, although available, was not incorporated until the scoring; therefore, the cardiovascular component represents a simplified version of the standard SOFA definition. Neurological status was assessed using the GCS, scored as follows: GCS 15 = 0; 13–14 = 1; 10–12 = 2; 6–9 = 3; <6 = 4. Renal function was scored using the highest serum creatinine in mg/dL (<1.2 = 0; 1.2-1.9 = 1; 2.0-3.4 = 2; 3.5-4.9 = 3; ≥5.0 = 4). Where a component variable was unavailable in the EMRs, it was assumed to be within the normal range and assigned a score of zero. This approach may underestimate the degree of organ dysfunction and represents a potential source of bias.

#### 2.5.2 APACHE II score

The modified APACHE II score was calculated by summing three components: an acute physiological score, age points, and chronic health points (20). For the physiological component, body temperature was scored as follows: 36.1–38.4 °C = 0; 34.0–36.0 °C or 38.5–38.9 °C = 1; 32.0–33.9 °C = 2; 30.0–31.9 °C or 39.0–40.9 °C = 3; <30.0 °C or ≥41.0 °C = 4. MAP was scored as follows: 70–109 mmHg = 0; 50–69 or 110–129 mmHg = 2; 130–159 mmHg = 3; ≥160 mmHg = 4. Serum creatinine was scored as follows: 0.6–1.4 mg/dL = 0; 1.5–1.9 mg/dL = 2; 2.0–3.4 mg/dL = 3; ≥3.5 mg/dL = 4. Neurological status was incorporated as 15 minus the actual GCS score. Age points were assigned as follows: <45 years = 0; 45–54 years = 2; 55–64 years = 3; 65–74 years = 5; ≥75 years = 6. Chronic health points were assigned based on the presence of qualifying conditions—including cirrhosis, heart failure, respiratory failure, chronic obstructive pulmonary disease, end-stage renal disease, malignancy, leukemia, lymphoma, acquired immunodeficiency syndrome, or immunosuppression—identified through diagnosis text fields and binary comorbidity indicators in the EMRs. Surgical patients with a qualifying condition were assigned two chronic health points; medical patients were assigned five points. The maximum attainable score depends on the included variables, with higher values indicating greater illness severity and predicted mortality risk. Because several standard APACHE II physiological variables — including heart rate, respiratory rate, oxygenation parameters, arterial pH or bicarbonate, serum sodium, potassium, hematocrit, and white blood cell count — were not consistently available, a reduced-variable modified APACHE II score was constructed using the available parameters. A reduced-variable modified APACHE II score was constructed using the available parameters. This approach may underestimate illness severity and limits direct comparability with studies using the full APACHE II scoring system.

#### 2.5.3 Score-component availability

Because the modified SOFA and APACHE II scores have not been independently validated, they should be interpreted as pragmatic indices of admission severity and compared with the published literature only with caution. The degree of missingness differed by component. Source measurements were available for the great majority of patients for the renal (creatinine, 98.9%), respiratory (PaO_2_, 98.4%), neurological (GCS, 96.2%), and cardiovascular (MAP, 94.0%) domains, but were missing more frequently for the coagulation (platelets, 81.4% available) and hepatic (bilirubin, 63.4% available) domains. Where a component was not measured, it contributed zero points to the score (i.e., was treated as normal). Because unmeasured components were scored as normal, the modified scores are likely to underestimate the true organ-dysfunction burden; this conservative bias should be borne in mind when interpreting the severity-score results.

### 2.6 Statistical analysis

#### 2.6.1 Statistical tests used

All statistical analyses were performed using R statistical software. Continuous variables were assessed for normality using the Shapiro–Wilk test and visual inspection of histograms; as all continuous variables departed from normality, they are reported as median (interquartile range, IQR) and were compared between groups using the Mann–Whitney *U* test. Categorical variables are presented as frequencies and percentages, *n* (%).

Bivariate analysis was conducted for comparisons between groups (AKI vs. no AKI; prolonged vs. short ICU stay). Categorical variables were compared by using chi-square test when all expected cell frequencies were ≥5, and Fisher’s exact test when any expected cell frequency was <5.

All potential predictors were entered into univariate logistic regression analyses, yielding crude odds ratios (OR) with 95% confidence intervals (CIs). Variables meeting *p* ≤ 0.20 in univariate analysis or considered clinically relevant were entered into multivariable logistic regression models. Statistical significance was set at *p* < 0.05 (two-tailed).

#### 2.6.2 Variable selection for multivariable regression models

The number of predictors that could be entered was limited by the number of outcome events, to preserve approximately 10 events per variable and reduce overfitting. For the AKI model, only 28 events were available (and fewer in covariate-complete records); thus, a maximum of three predictors was retained; where more candidates met the screening criterion than could be accommodated, selection prioritized the variables with the strongest univariate associations and greatest clinical relevance. For this reason, although infection and SOFA met the screening threshold for AKI (univariate *p* = 0.106 and 0.099, respectively), they were not entered into the AKI model, whereas hypertension, admission glucose, and APACHE II score, which showed stronger univariate associations, were retained. The number of predictors entered was constrained with reference to the number of events per variable to limit overfitting (21). In the ICU LOS multivariable model, 47 events were available; thus, four predictors were retained. Therefore, infection, admission sodium levels, AKI occurrence, and admission potassium levels were retained. Adjusted ORs (aOR) with 95% CIs were reported.

#### 2.6.3 Missing data

Regression analyses used complete-case methods; no values were imputed. Missingness was low (<6%) for demographic, comorbidity, precipitating factor, and most admission laboratory variables (sodium, potassium, and creatinine), but higher for laboratory plasma glucose in mmol/L (22.4%), HbA1c (36.1%), and ICU length of stay (10.4%). Point-of-care glucose (mg/dL) was more completely recorded (9.3% missing) than laboratory plasma glucose. Because some variables, particularly HbA1c and laboratory glucose, are unlikely to be missing completely at random, the potential for selection bias is acknowledged, and analyses involving these variables should be interpreted accordingly.

#### 2.6.4 Sensitivity analysis

To address the possibility of overfitting in the multivariable AKI model, we performed additional sensitivity analyses. Although AKI occurred in 28 patients overall, the multivariable model was estimated on complete cases for all predictors; because admission glucose was missing in a proportion of patients, the model was based on 139 patients with 19 AKI events, corresponding to approximately 6.3 events per variable. To assess robustness under this constraint, we performed sensitivity analyses using parsimonious models that entered admission glucose alone (19 events per variable) and admission glucose with the APACHE II score (~9.5 events per variable), thereby increasing the number of events per variable relative to the primary model.

For the prolonged-ICU-stay outcome, the multivariable model was repeated across length-of-stay thresholds of ≥2, ≥3, ≥4, and ≥5 days, the ≥3-day threshold being the pre-specified primary definition.

## 3 Results

### 3.1 Cohort characteristics

A total of 183 adult patients admitted to the ICU with DKA were included in this study. These represented first ICU admissions between July 2012 and December 2024. Sample sizes varied slightly across analyses due to missing data for certain variables.

As shown in **Table 1**, the cohort had a median age of 33.0 years (IQR, 24–53) and was predominantly female (60.7%). Most patients had type 1 diabetes (78.1%) with a median duration of 7.0 years (IQR, 2–12). The most common comorbidity was hypertension (24.6%), followed by IHD (7.1%) and CKD (3.8%). Various precipitating factors of DKA were also noted with infection identified as the most common (35%) followed by insulin non-adherence (20.2%) and new-onset diabetes (14.8%).

**Table 1:** Baseline characteristics and treatment.

Variable*	Total (*n* = 183)
Demographics	
Age, years, median (IQR)	33.0 (24.0–53.0)
Female, *n* (%)	111 (60.7)
Male, *n* (%)	72 (39.3)
Diabetes characteristics	
Type 1 DM, *n* (%)	143 (78.1)
Type 2 DM, *n* (%)	40 (21.9)
Duration of DM, years, median (IQR) (*n* = 124)	7.0 (2.0–12.0)
Comorbidities	
Hypertension, *n* (%)	45 (24.6)
IHD, *n* (%)	13 (7.1)
CKD, *n* (%)	7 (3.8)
Number of comorbidities, median (IQR)	1.0 (1.0–2.0)
Precipitating factors	
Infection, *n* (%)	64 (35.0)
Insulin omission/non-adherence, *n* (%)	37 (20.2)
New-onset diabetes, *n* (%)	27 (14.8)
Severity scores at admission	
SOFA score, median (IQR)	2 (1–3)
APACHE II score, median (IQR)	2 (2–5)
Vital signs at admission	
Systolic BP, mmHg, median (IQR) (*n* = 172)	123 (110–135)
Heart rate, bpm, median (IQR) (*n* = 172)	98 (84.25–111)
Temperature, °C, median (IQR) (*n* = 177)	36.9 (36.6–37.0)
SpO2, %, median (IQR) (*n* = 178)	97 (95–98)
Laboratory values at admission	
Point-of-care glucose, mg/dL, median (IQR) (*n* = 166)	400 (310–500)
Laboratory plasma glucose, mmol/L, median (IQR) (*n* = 142)	16.2 (12.6–21.2)
HbA1c, %, median (IQR) (*n* = 117)	11.3 (9.8–13.4)
Potassium, mmol/L, median (IQR) (*n* = 179)	4.2 (3.8–4.7)
Sodium, mmol/L, median (IQR) (*n* = 179)	137.5 (134–140)
Creatinine, µmol/L, median (IQR) (*n* = 181)	72.0 (56.8–100.5)
Treatment	
IV insulin infusion, *n* (%) (*n* = 147)	135 (91.8)
Duration of IV insulin, h, median (IQR) (*n* = 103)	48 (24–72)
Potassium supplementation, *n* (%) (*n* = 149)	102 (68.5)
Bicarbonate therapy, *n* (%) (*n* = 149)	19 (12.8)
Vasopressor use, *n* (%) (*n* = 162)	3 (1.8)

*For each variable, *n* = 183 unless otherwise stated. For variables with missing data, the available-case denominator is given in parentheses (*n*) and percentages are calculated accordingly; variables without an indicated *n* were complete.

IQR, interquartile range; DM, diabetes mellitus; IHD, ischemic heart disease, CKD, chronic kidney disease; SOFA, Sequential Organ Failure Assessment; APACHE, Acute Physiology and Chronic Health Evaluation; BP, blood pressure; SpO2, oxygen saturation; IV, intravenous.

The median SOFA score was 2 (IQR, 1–3) and the median for APACHE score was 2 (IQR, 2–5). Most patients required intravenous insulin infusion (91.8%) and approximately two-thirds required potassium supplementation (68.5%). The laboratory findings at ICU admission are also shown in **Table 1**.

### 3.2 Clinical outcomes

In this cohort, AKI occurred in 28 of 176 evaluated patients (15.9%). The median ICU length of stay was 2.0 days (IQR, 1.0–3.0; *n* = 164), and 47 patients (28.7%) experienced a prolonged ICU stay (≥3 days), as per the pre-specified study definition. Thirteen patients (7.1%) were readmitted within 90 days of the initial hospitalization and eight (4.4%) died within 90 days (**Table 2**).

**Table 2:** Clinical outcomes.

Outcome*	Total (*n* = 183)
ICU outcomes	
AKI (*n* = 176), *n* (%)	28 (15.9)
ICU length of stay, days, median (IQR) (*n* = 164)	2.0 (1.0–3.0)
Prolonged ICU stay (≥3 days), *n* (%) (*n* = 164)	47 (28.7)
Readmission	
Any readmission within 90 days, *n* (%)	13 (7.1)
Mortality	
90-day all-cause mortality, *n* (%)	8 (4.4)

* For each variable, *n* = 183 unless otherwise stated. For variables with missing data, the available-case denominator is given in parentheses (*n*) and percentages are calculated accordingly; variables without an indicated *n* were complete.

ICU, intensive care unit; AKI, acute kidney injury.

### 3.3 Bivariate analysis of AKI and ICU stay predictors

Bivariate comparisons between patients with and without AKI, and between those with short and prolonged ICU length stays are presented in **Table 3**. Hypertension (*p* = 0.013), elevated admission laboratory glucose in mmol/L (*p* = 0.037), and higher APACHE II score (*p* = 0.002) were significantly associated with AKI occurrence. Patients who developed AKI also had a significantly higher rate of 90-day readmission (*p* = 0.002).

**Table 3:** Bivariate analysis of predictors of AKI and prolonged ICU stay in patients with DKA.

Variable	No AKI (*n* = 148)	AKI (*n* = 28)	*p*-value	Short ICU stay (*n* = 117)	Prolonged ICU stay (*n* = 47)	*p*-value
Demographics						
Age, median (IQR)	30 (23–51)	43.5 (26–54)	0.149	32 (22–53)	34 (24–54)	0.550
Male sex, *n* (%)	55 (37.2)	14 (50.0)	0.202	48 (41.0)	18 (38.3)	0.747
Comorbidities						
Type 1 diabetes	119 (80.4)	22 (78.6)	0.824	89 (76.1)	37 (78.7)	0.716
Hypertension, *n* (%)	31 (20.9)	12 (42.9)	**0.013**	29 (24.8)	9 (19.1)	0.439
CKD, *n* (%)	4 (2.7)	2 (7.1)	0.244	4 (3.4)	1 (2.1)	1.000
IHD, *n* (%)	10 (6.8)	1 (3.6)	1.000	10 (8.6)	2 (4.3)	0.512
Precipitating factors						
Infection, *n* (%)	50 (33.8)	14 (50.0)	0.102	33 (28.2)	31 (66.0)	**<0.001**
Insulin non-adherence, *n* (%)	30 (20.3)	5 (17.9)	0.769	28 (23.9)	9 (19.1)	0.508
New-onset diabetes, *n* (%)	24 (16.2)	3 (10.7)	0.577	17 (14.5)	10 (21.3)	0.292
Laboratory values						
Laboratory plasma glucose (mmol/L), median (IQR) †‡	15.5 (12.6–20.3)	18.9 (15.2–27.0)	**0.037**	15.4 (12.6–20.9)	16.7 (12.0–21.9)	0.602
Point-of-care glucose (mg/dL), median (IQR) † ‡	390 (303–500)	420 (351–524)	0.165	400 (319–500)	397 (301–520)	0.552
Potassium mmol/L, median (IQR) †‡	4.2 (3.7–4.7)	4.2 (3.7–5.0)	0.858	4.3 (3.9–4.8)	4.1 (3.7–4.5)	**0.027**
Sodium mmol/L, median (IQR) †‡	137.0 (134.0–139.0)	136.0 (129.5–141.0)	0.520	136.0 (133.0–138.0)	139.0 (136.0–142.0)	**<0.001**
Severity scores						
Total SOFA score, median (IQR)	2 (1–3)	2.5 (1–5)	0.240	2 (1–3)	3 (2–4)	0.316
Total APACHE II score, median (IQR)	2 (1.2–5)	5.5 (2–8)	**0.002**	2 (2–5)	3 (2–6)	0.609
Clinical outcomes						
AKI‡, *n* (%)	—	—	—	10 (9.1)	14 (29.8)	**0.001**
90-day readmission	7 (4.7)	6 (21.4)	**0.002**	7 (6.0)	5 (10.6)	0.301

† Sample sizes for patients with AKI recorded varied due to missing data [sum to 176 rather than 183 because AKI variable was missing for 7 patients (3.8%)]: Laboratory plasma glucose (mmol/L) *n* = 139, 19 AKI cases; Point-of-care glucose (mg/dL) *n* = 159, 24 AKI cases; Potassium and sodium *n* = 175, 28 AKI cases.

‡ Sample sizes for patients with ICU stay recorded varied due to missing data [sum to 164 rather than 183 because ICU length of stay was missing for 19 patients (10.4%)]: AKI *n* = 157, 47 in long ICU stay group; Laboratory plasma glucose (mmol/L) *n* = 136, 39 long ICU stay; Point-of-care glucose (mg/dL) *n* = 157, 47 long ICU stay; Potassium and sodium *n* = 160, 47 long ICU stay.

IHD, ischemic heart disease, CKD, chronic kidney disease; SOFA, Sequential Organ Failure Assessment; APACHE, Acute Physiology and Chronic Health Evaluation.

Bold values indicate statistical significance (p < 0.05).

Infection, as a precipitating factor (*p* < 0.001), AKI occurrence (*p* = 0.001), and admission sodium and potassium levels (*p* < 0.001, and *p* = 0.027, respectively) were significantly associated with prolonged ICU stay.

### 3.4 Predictors of AKI

In the univariate logistic regression model, hypertension (OR 2.83; 95% CI 1.21–6.60; *p* = 0.016), admission glucose per mmol/L (OR 1.06; 95% CI 1.01–1.11; *p* = 0.013), and APACHE II score per point (OR 1.20; 95% CI 1.07–1.35; *p* = 0.002) were significantly associated with AKI. In the multivariable model, only admission glucose remained an independent predictor (aOR 1.05; 95% CI 1.00–1.10; *p* = 0.034). Hypertension and APACHE II score did not retain statistical significance after adjustment (**Table 4**).

**Table 4:** Predictors of AKI: Univariate and multivariable logistic regression.

Variable	OR (95% CI)	*p*-value	aOR (95% CI)	*p*-value
Demographics				
Age (per year)	1.01 (0.99–1.03)	0.298	--	--
Male sex	1.69 (0.75–3.81)	0.205	--	--
Comorbidities				
Type 2 diabetes vs. type 1	1.12 (0.42–3.01)	0.824	--	--
Hypertension (Yes vs. No)	2.83 (1.21–6.60)	**0.016**	1.15 (0.36–3.68)	0.811
CKD (Yes vs. No)	2.77 (0.48–15.90)	0.253	--	--
IHD (Yes vs. No)	0.51 (0.06–4.16)	0.530	--	--
Precipitating factors			--	--
Infection (Yes vs. No)	1.96 (0.87–4.43)	0.106	--	--
Insulin non-adherence (Yes vs. No)	0.86 (0.30–2.44)	0.769	--	--
New-onset diabetes (Yes vs. No)	0.62 (0.17–2.22)	0.462	--	--
Laboratory values			--	--
Laboratory plasma glucose (per mmol/L)	1.06 (1.01–1.11)	**0.013**	1.05 (1.00–1.10)	**0.034**
Point-of-care glucose (per mg/dL)	1.00 (1.00–1.01)	0.060	--	--
Potassium (per mmol/L)	1.26 (0.70–2.26)	0.442	--	--
Sodium (per mmol/L)	0.98 (0.92–1.05)	0.612	--	--
Severity scores			--	--
Total SOFA score (per point)	1.23 (0.96–1.57)	0.099	--	--
Total APACHE II score (per point)	1.20 (1.07–1.35)	**0.002**	1.12 (0.97–1.30)	0.116

OR, odds ratio; aOR, adjusted odds ratio; CI, confidence interval. aOR reported only for variables entered into the multivariable model. IHD, ischemic heart disease, CKD, chronic kidney disease; SOFA, Sequential Organ Failure Assessment; APACHE, Acute Physiology and Chronic Health Evaluation.

Bold values indicate statistical significance (p < 0.05).

**Figure 1:**
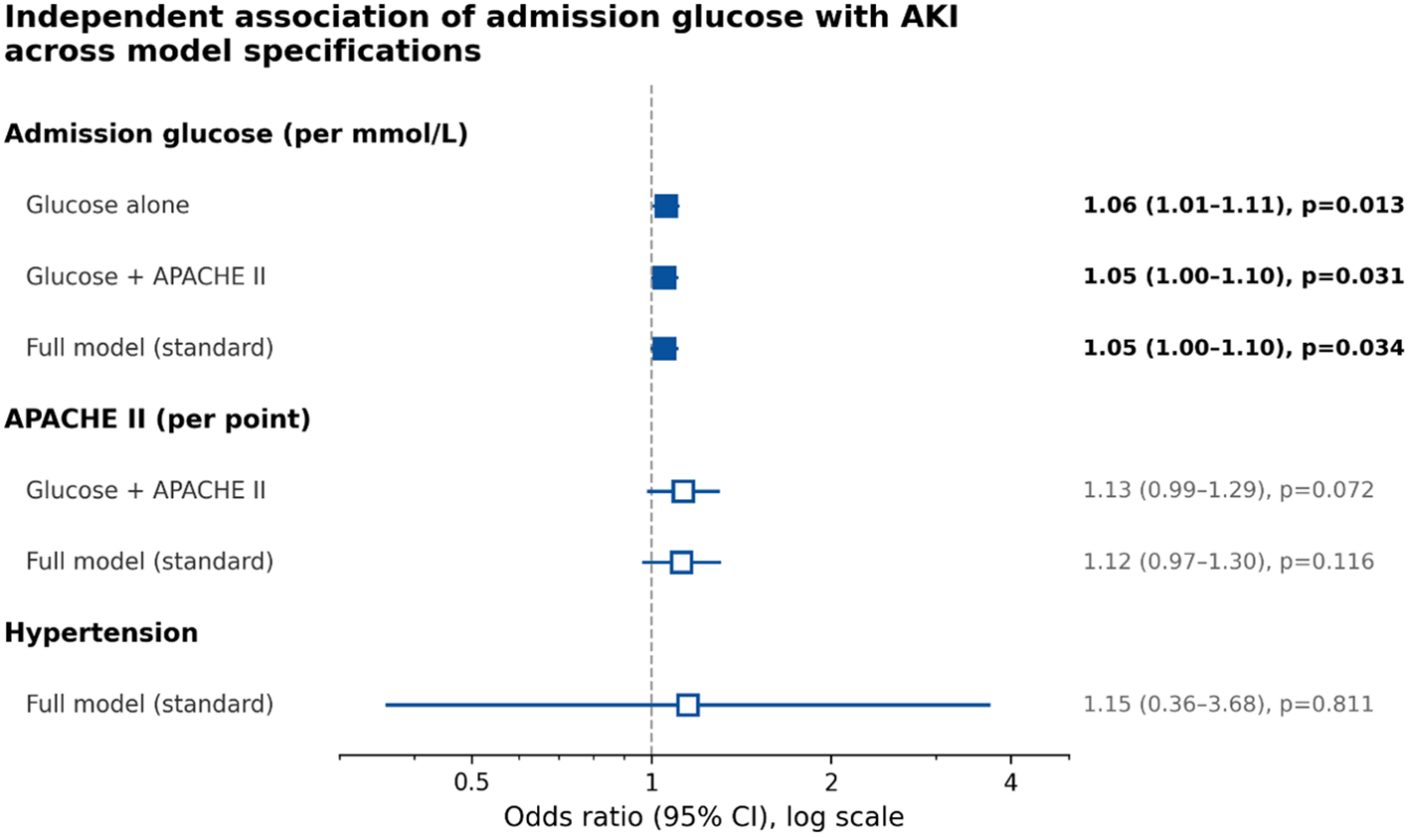
Independent association of glucose with AKI across model specifications. Forest plot showing odds ratios (ORs) with 95% confidence intervals (CIs) for predictors of acute kidney injury (AKI). Admission glucose was entered alone, with the APACHE II score, and in the full primary model together with hypertension and APACHE II. All models were fitted in the same cohort (*n* = 139; 19 AKI events). Filled squares denote statistically significant associations (*p* < 0.05) and open squares indicate non-significant associations; the dashed vertical line marks the null value (OR = 1), and the *x*-axis is on a logarithmic scale. Glucose remained an independent predictor of AKI of essentially constant magnitude across all three specifications, whereas APACHE II and hypertension were not independently associated in any specification, indicating that the glucose–AKI association is robust to model choice.

The association between admission glucose and AKI was stable across sensitivity analyses. The association persisted in parsimonious models entering glucose alone (OR 1.06, 95% CI 1.01–1.11; *p* = 0.013) and glucose with APACHE II (adjusted OR 1.05, 95% CI 1.00–1.10; *p* = 0.031), each with a higher number of events per variable than the primary model. The consistency of the estimate across these models indicates that the glucose–AKI association is not an artifact of overfitting (**Figure 1**).

### 3.5 Predictors of prolonged ICU stay

In univariate logistic regression analysis, infection (OR 4.93, 95% CI 2.39–10.19, *p* < 0.001), higher admission sodium levels (OR 1.09 per mmol/L, 95% CI 1.03–1.16, *p* = 0.004), lower potassium levels (OR 0.53 per mmol/L, 95% CI 0.31–0.93, *p* = 0.026), and AKI occurrence (OR 4.24, 95% CI 1.72–10.45, *p* = 0.002) were significantly associated with prolonged ICU stay. In the multivariable logistic regression model, all four variables—infection (aOR 4.00, 95% CI 1.83–8.76, *p* < 0.001), admission sodium level (aOR 1.07 per mmol/L increase, 95% CI 1.00–1.14, *p* = 0.039), admission potassium (aOR 0.52, 95% CI 0.28–0.97, *p* = 0.040), and AKI occurrence (aOR 4.88, 95% CI 1.73–13.77, *p* = 0.003)—remained independently significant (**Table 5**).

**Table 5:** Predictors of longer ICU stay—Univariate and multivariable logistic regression.

Variable	OR (95% CI)	*p*-value	aOR (95% CI)	*p*-value
Demographics				
Age (per year)	1.00 (0.99–1.02)	0.701	--	--
Male sex vs. Female	0.89 (0.45–1.79)	0.747	--	--
Severity scores				
Total SOFA score (per point)	1.12 (0.91–1.38)	0.297	--	--
Total APACHE II score (per point)	1.04 (0.94–1.15)	0.506	--	--
Outcomes				
AKI (Yes vs. No)	4.24 (1.72–10.45)	**0.002**	4.88 (1.73–13.77)	**0.003**
Comorbidities				
Hypertension (Yes vs. No)	0.72 (0.31–1.66)	0.440	--	--
CKD (Yes vs. No)	0.61 (0.07–5.64)	0.667	--	--
IHD (Yes vs. No)	0.48 (0.10–2.26)	0.350	--	--
Type 2 diabetes vs. type 1	0.86 (0.38–1.95)	0.716	--	--
Precipitating factors				
Infection (Yes vs. No)	4.93 (2.39–10.19)	**<0.001**	4.00 (1.83–8.76)	**<0.001**
Insulin omission (Yes vs. No)	0.75 (0.32–1.75)	0.509	--	--
New-onset diabetes (Yes vs. No)	1.59 (0.67–3.78)	0.295	--	--
Laboratory values at admission				
Laboratory plasma glucose (per mmol/L)	1.01 (0.96–1.05)	0.775	--	--
Point-of-care glucose (per mg/dL)	1.00 (1.00–1.00)	0.989	--	--
Potassium (per mmol/L)	0.53 (0.31–0.93)	**0.026**	0.52 (0.28–0.97)	**0.040**
Sodium (per mmol/L)	1.09 (1.03–1.16)	**0.004**	1.07 (1.00–1.14)	**0.039**

OR, odds ratio; aOR, adjusted odds ratio; CI, confidence interval. aOR reported only for variables entered into the multivariable model. IHD, ischemic heart disease, CKD, chronic kidney disease; SOFA, Sequential Organ Failure Assessment; APACHE, Acute Physiology and Chronic Health Evaluation.

Bold values indicate statistical significance (p < 0.05).

**Figure 2:**
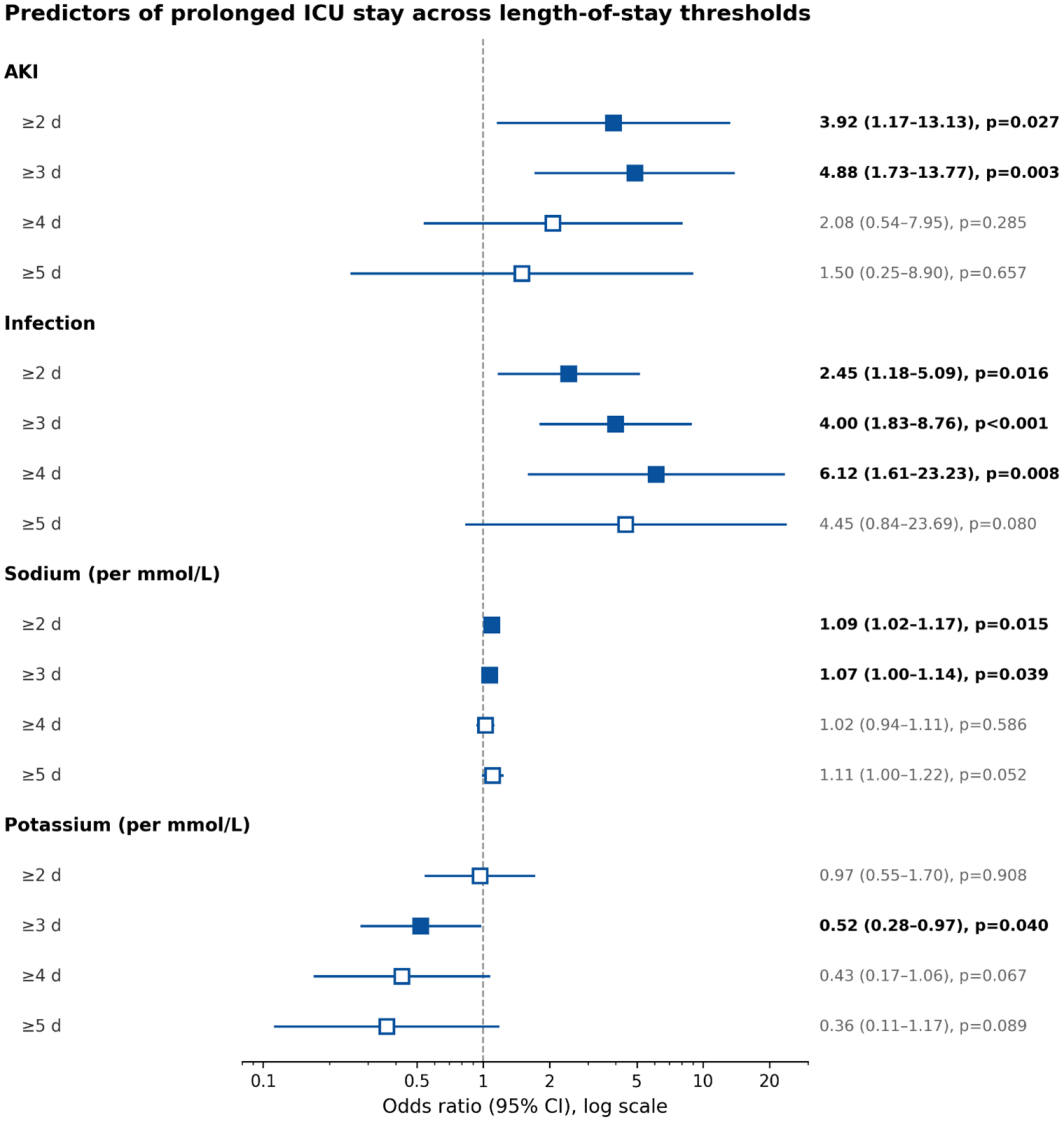
Predictors of prolonged ICU stay across length-of-stay thresholds. Forest plot of adjusted odds ratios (aORs) with 95% confidence intervals (CIs) for the four predictors of the primary multivariable model—AKI, infection, admission sodium, and admission potassium—with the prolonged-stay outcome defined at four thresholds: ≥2, ≥3 (the pre-specified primary definition), ≥4, and ≥5 days. Models were fitted in the same cohort (*n* = 164); the number of outcome events at each threshold is 96, 47, 15, and 9, respectively. Filled squares denote statistically significant associations (*p* < 0.05) and open squares indicate non-significant associations. The dashed vertical line marks the null value (OR = 1). AKI, sodium, and infection remained independently associated with prolonged stay at the adequately powered ≥2- and ≥3-day thresholds (infection also at ≥4 days), whereas the association with admission potassium was significant only at the ≥3-day threshold. The attenuation of effects at ≥4 and ≥5 days reflects the small number of events (≈3.8 events per variable) and consequent loss of statistical power rather than a true reversal of effect.

In sensitivity analyses, the primary model (AKI, infection, admission sodium, and potassium) was repeated across length-of-stay thresholds of ≥2, ≥3, ≥4, and ≥5 days. AKI, sodium, and infection were more consistent predictors (**Figure 2**). At ≥2 days (96 events), only three covariates were independently associated with prolonged stay: AKI (aOR 3.92, 95% CI 1.17–13.13, *p* = 0.027), infection (aOR 2.45, 95% CI 1.18-5.09, *p* = 0.016), and higher sodium (1.09 per mmol/L, 95% CI 1.02–1.17, *p* = 0.015). At the primary ≥3-day threshold (47 events), AKI, infection, and sodium remained independently associated, and admission potassium was also significant (aOR 0.52, 95% CI 0.28–0.97, *p* = 0.040). At ≥4 and ≥5 days, the models were underpowered (15 and 9 events; ≈3.8 and ≈2.3 events per variable); only infection retained significance at ≥4 days (aOR 6.12, 95% CI 1.61–23.23, *p* = 0.008), with AKI and sodium attenuating (*p* = 0.285 and 0.586, respectively), consistent with loss of power rather than a true reversal. Overall, higher sodium, AKI, and infection were robust across the adequately powered thresholds, whereas potassium was significant only at the ≥3-day definition.

## 4 Discussion

In this 12-year retrospective cohort of ICU-admitted patients with DKA at a Jordanian tertiary hospital, AKI developed in approximately one in six patients and was independently associated with prolonged ICU stay. Furthermore, AKI was significantly associated with 90-day hospital readmission, underscoring the persistent morbidity burden of renal injury in this population. Admission hyperglycemia was the sole independent predictor of AKI on multivariable analysis, while infection, AKI, lower admission potassium, and elevated admission sodium were independently associated with prolonged ICU stay.

Published cohorts report AKI incidences in DKA that vary more than fourfold — from 15.9% in the present cohort to 64.2% in hospitalized children (**Table 6**) — and this heterogeneity appears to reflect differences in population, care setting, and methodology rather than a true difference in renal susceptibility. Age shows a consistent gradient: pediatric cohorts report the highest rates [Hursh 2017, 64.2% (12); Huang 2022, 47% (11) of episodes], whereas our young, predominantly type 1 diabetic adults and the comparably young Bangladeshi cohort [Rahim 2018 (22), mean age 37.6 years] report among the lowest (15.9% and 29.5%, respectively). Care setting and illness severity contribute further: a dedicated severe-DKA ICU cohort [Orban 2014, 50% (7)] and hospitalized cohorts with an older case mix [Chen 2020, 54.8% (9)] report higher rates, and the MIMIC-IV analysis [Hua 2024 (8)] demonstrates the severity dependence directly, with AKI rising from 14.2% to 62.3% across SOFA strata. Methodological factors—differing AKI criteria (KDIGO, RIFLE, and AKIN), inclusion of all hospitalized versus ICU-only patients, and the choice of baseline creatinine—shift the apparent incidence still further. A US national study of 464,057 DKA hospitalizations that identified AKI from administrative diagnostic codes reported an intermediate overall rate of 37.8% that climbed from 28.2% to 46.3% across the decade, illustrating how ascertainment method and calendar period—not renal biology alone—shape the reported incidence (10). Viewed in this context, the relatively low AKI rate in our cohort is consistent with its young, predominantly type 1 diabetic composition and with our conservative, admission-anchored ascertainment of AKI, rather than indicating a genuinely lower susceptibility to renal injury.

**Table 6:** Acute kidney injury in diabetic ketoacidosis across selected studies.

Study	Country	Setting	*N*	AKI definition	AKI (%)‡	Notable features	Reference
Current study	Jordan	Adult ICU; first admission	183	Serum creatinine, KDIGO 2012	15.9	Young cohort (median 33 years), ~78% type 1 DM; AKI ascertained from post-admission creatinine change, so AKI present on arrival was not captured, yielding a conservative estimate	--
Orban 2014	France	Adult ICU; severe DKA	94	Serum creatinine, RIFLE	50.0	Severe-DKA ICU cohort; AKI defined at admission and largely pre-renal/volume-responsive, resolving with fluid resuscitation	(7)
Chen 2020	China	Hospitalized DKA (ward and ICU)	179	Serum creatinine, KDIGO 2012	54.8	Older case mix; higher admission glucose and older age were independent predictors; AKI linked to worse long-term renal outcomes	(9)
Rahim 2018	Bangladesh	Adult; tertiary referral hospital	200	Serum creatinine, AKIN	29.5	Similar young mean age (37.6 years); infection the commonest precipitant (40.5%); AKI more frequent with sepsis and pancreatitis.	(22)
Hua 2024	USA	Adult ICU (MIMIC-IV database)	626	Serum creatinine, KDIGO	33.5*	AKI incidence rose steeply with organ-failure severity (14.2% at SOFA ≤3 vs. 62.3% at SOFA >3), illustrating dependence on ICU case mix and severity	(8)
Tian 2026	USA	Hospitalized DKA (nationwide inpatient sample)	464,057	ICD-9/10 diagnostic codes	37.8	Largest cohort to date; AKI ascertained from administrative codes rather than measured creatinine; prevalence rose from 28.2% (2010) to 46.3% (2019); AKI linked to higher mortality, dialysis/ventilation, longer stay, and ~50% higher costs	(10)
Hursh 2017	Canada	Children hospitalized for DKA (type 1 DM)	165	Serum creatinine, KDIGO	64.2	Landmark pediatric study; AKI associated with greater acidosis, higher heart rate and corrected sodium (volume depletion); most cases resolved with rehydration	(12)
Huang 2022	USA	Children with type 1 DM (DKA episodes)	2,345	Serum creatinine, KDIGO	47†	AKI during DKA in ~47% of episodes; recurrent DKA-associated AKI linked to later microalbuminuria, signaling long-term renal risk	(11)

AKI = acute kidney injury; DKA = diabetic ketoacidosis; DM = diabetes mellitus; ICU = intensive care unit; KDIGO = Kidney Disease: Improving Global Outcomes; RIFLE = Risk, Injury, Failure, Loss, End-stage; AKIN = Acute Kidney Injury Network; SOFA = Sequential Organ Failure Assessment.

* Hua et al. report AKI stratified by SOFA strata rather than as a single overall figure; the 33.5% range is calculated from the study data.

† Proportion of DKA episodes (560 of 1,190 analyzed episodes among 2,345 children), not of patients.

‡ Studies differ in AKI criteria (KDIGO, RIFLE, and AKIN), ascertainment method (measured serum creatinine vs. administrative diagnostic codes), care setting (ICU only vs. all hospitalized), and baseline-creatinine conventions; reported AKI percentages are therefore not directly comparable.

Hypertension, elevated glucose levels, and higher APACHE scores were associated in univariate analysis with AKI in our study. However, after adjustment for potential confounders, only admission laboratory glucose remained an independent predictor of AKI. Hypertension and APACHE II score were significant in univariate analysis but did not retain independent associations, suggesting that their apparent relationship with AKI is mediated through glycemic severity or confounded by other variables. This finding aligns with previous studies that identified hyperglycemia as an independent predictor of AKI in patients with DKA from China (OR 1.087; 95% CI: 1.034–1.142, *p* = 0.001) (9) and France (OR 1.101; 95% CI: 1.039–1.166; *p* < 0.01) (7). These findings reinforce the current international guideline recommendations for prompt fluid resuscitation and correction of hyperglycemia to mitigate physiologic derangements and support renal perfusion in hyperglycemic crises (16, 23), which may thereby reduce the risk of AKI.

Because of the retrospective, observational design, these associations should be interpreted as associations rather than as established causal relationships. Severe hyperglycemia may reflect more severe dehydration and osmotic stress, both of which contribute to renal hypoperfusion and tubular injury (16). The consistency of the glucose–AKI association with prior reports, together with its robustness in parsimonious models, supports its plausibility, but prospective studies incorporating direct markers of dehydration and illness severity (for example, fluid balance, lactate, and validated severity scores) are needed to confirm the relationship and clarify its direction.

Approximately one-third of patients required an ICU stay of ≥3 days. AKI was independently associated with more than a fourfold increase in the odds of prolonged ICU stay. This observation is consistent with established literature that shows that renal dysfunction is linked to delayed recovery, complex fluid management, and the need for extended monitoring in critical care (17, 24). In a retrospective analysis of over 5,000 patients who were followed in the ICU, the development of AKI was significantly associated with prolonged ICU-LOS [OR (95% CI): 1.807 (1.434–2.277), *p* < 0.001] (25). Another cohort study reported that AKI incidence, stage, and duration were all significantly associated with prolonged ICU and hospital LOS (*p* < 0.01), discharge destination other than home (*p* < 0.05), and mortality (*p* < 0.01) in patients admitted to the ICU after severe trauma (26). This association could be related to the requirement of dialysis for a few days, weeks, or even months after an AKI, resulting in prolonged ICU or hospital stay (27).

Infection was a major contributor to the increase in ICU stay in our study. It is a recognized trigger for DKA as it increases systemic inflammation, worsens insulin resistance, and leads to hemodynamic instability (28, 29). Together, these factors likely contribute to prolonged metabolic derangements and delayed clinical improvement (26, 30). According to a case–control study conducted in nine Australian public hospitals, hospital-acquired bloodstream infections were associated with longer LOS in the ICU, highlighting the importance of preventing infections in order to reduce the utilization of resources and improve patient outcomes (31, 32). Another cohort study conducted in India found a significant relationship between LOS in ICU and nosocomial infections [OR (95% CI): 1.25 (1.07–1.46), *p* = 0.0005] (33).

Higher admission sodium levels were also found to be an independent predictor of extended ICU stay in our study. Similar findings were reported in a large multicenter cohort study involving over 200,000 participants from 344 ICUs across the United States (34). The study found that ICU-acquired hypernatremia (serum sodium >149 mEq/L) was independently associated with a 28% increase in ICU LOS [rate ratio (RtR) 1.28; 95% CI: 1.26–1.30]. In addition, the RtR increased with increasing severity of hypernatremia. Elevated sodium at presentation may reflect severe volume depletion and hyperosmolar states at presentation (34, 35). Controlled and gradual reduction in serum sodium levels is essential to avoid neurologic sequelae that may necessitate longer ICU monitoring (35, 36). In contrast, lower admission potassium was independently associated with prolonged ICU stay. Potassium disturbances are characteristic of DKA: serum potassium is often normal or high at presentation despite marked total-body depletion, and hypokalemia commonly emerges during insulin therapy and correction of acidosis (37). A relatively low admission potassium may therefore mark more severe total-body depletion, identifying patients who need closer monitoring and longer ICU care. As this association reached significance only at the primary ≥3-day threshold, it should be regarded as hypothesis-generating. Although severity scores such as SOFA and APACHE are recognized predictors of mortality in ICU populations, they did not independently predict prolonged ICU stay after adjustment in our study (8, 38). Their predictive ability for duration of ICU stay may vary depending on patient population and the overall clinical burden of the disease (39). According to a large multicenter, retrospective cohort study of critically ill patients with COVID-19 admitted to 16 hospitals in Texas, it was found that ICU scores such as the Simplified Acute Physiology Score (SAPS) II, APACHE II, SOFA score, and 4C Mortality scores were all significant predictors of mortality. However, they were not significant predictors of ICU LOS (40), suggesting that despite being effective in predicting mortality, it appears that these scores might have inconsistent ability to predict duration outcomes such as LOS, depending on the population and the disease burden.

The 90-day mortality rate from DKA has substantially declined in recent years as observed in our study, reflecting improvements in standardized treatment protocols (41). The 4.4% 90-day mortality in this cohort is lower than in some published series, likely reflecting the younger patient population and the predominance of type 1 diabetes. Non-survivors were older and had higher APACHE II scores at admission; however, formal mortality prediction modeling was not conducted owing to the small number of events. During the period from 2000 to 2014, a report from the United States reported a consistent decline in in-hospital case-fatality rates among patients with DKA, despite a rise in DKA hospitalizations among all age groups (41). Furthermore, a previous review noted that appropriate treatment of DKA reduced mortality rates to <1%, highlighting the importance of acute management strategies in reducing mortality rates in patients with DKA (4, 41).

AKI showed a significant association with readmission. Prior studies have demonstrated that in-hospital AKI increases the risk of subsequent morbidity and rehospitalization (42), potentially due to incomplete renal recovery or ongoing metabolic vulnerability. Hospital-acquired AKI was independently associated with higher likelihood of 30-day hospital readmission (OR, 1.21; 95% CI, 1.08–1.36), which persisted at 60 (OR, 1.15; 95% CI, 1.03–1.27) and 90 days (adjusted OR, 1.13; 95% CI, 1.02–1.25), according to a retrospective cohort study (43). Another large retrospective analysis reported that patients who developed AKI had 62% higher risk of being readmitted at 90 days after discharge for any cause compared with those without AKI [hazard ratio (HR) (95% CI): 1.62 (1.60–1.65)] (44).

Timely recognition of patients at increased risk for AKI and prolonged ICU stay is essential, although early detection may be limited by reliance on serum creatinine alone. Admission hyperglycemia, infection status, and serum sodium levels represent readily obtainable clinical variables that may inform diabetes care delivery in the critical-care setting and assist in early risk stratification of patients with diabetes. Integrating these variables into clinical assessment at the time of ICU admission could facilitate early, diabetes-focused interventions, including aggressive fluid resuscitation and glycemic management for severe hyperglycemia, prompt antimicrobial therapy and source control for infection, and careful planning of sodium correction. Such risk-based management plans grounded in the principles of inpatient diabetes care have been associated with improved clinical outcomes and more efficient ICU resource utilization in critically ill populations with hyperglycemic crises (36, 45).

Given the increasing burden of DKA admissions globally (41), identifying modifiable outcomes of complications remains an important objective in clinical care.

Strengths of this study include its long observation period (12 years), standardized KDIGO-based AKI definition using serum creatinine criteria (46), and the use of event per variable-guided variable selection in regression modeling to minimize overfitting (47, 48). This study also provides data from a Middle Eastern ICU, contributing to a region that is underrepresented in DKA literature.

Several limitations must be acknowledged. First, the retrospective, single-center design restricts the ability to establish causality and generalizability. Second, residual confounding and missing laboratory data for key variables such as lactate, and anion gap, may have introduced selection bias. Third, urine output data were not routinely available and could not be incorporated into AKI staging, potentially leading to underestimation of AKI incidence and severity. Fourth, long-term renal outcomes beyond 90 days were not available. Fifth, because several physiological variables required to compute the standard SOFA and APACHE II scores were not consistently documented in the EMRs, modified versions of these scores were used. This approach may underestimate illness severity and limits direct comparability of our severity-of-illness data with studies employing the full scoring systems. Findings related to severity scores should therefore be interpreted with caution. An additional limitation concerns the AKI reference standard: in the absence of a reliably documented pre-admission creatinine, the admission value was used as the baseline. In patients who were already volume-depleted at presentation, this may have led some community-acquired AKI to be misclassified as baseline renal function, most likely underestimating the true AKI incidence; the low observed baseline creatinine and AKI prevalence should be interpreted with this in mind.

## 5 Conclusion

In this cohort of ICU-admitted patients with DKA, AKI developed in approximately one in six patients and was an independent predictor of prolonged ICU stay. Admission hyperglycemia emerged as the main independent predictor of AKI on multivariable analysis, whereas AKI, infection, and elevated sodium levels were independently associated with extended ICU hospitalization across length-of-stay thresholds. Early identification and focused management of these high-risk factors may improve clinical outcomes and optimize ICU resource utilization. Future multicenter prospective studies are warranted.

## Data Availability

The raw data supporting the conclusions of this article will be made available by the authors, without undue reservation.

## References

[1] Feingold KR Dhatariya K . Diabetic ketoacidosis. In: Feingold KR Ahmed SF Anawalt B Blackman MR Boyce A Chrousos G , editors. Endotext. MDText.com, Inc, South Dartmouth, MA: MDText.com, Inc (2026). Available online at: https://www.ncbi.nlm.nih.gov/books/NBK620701/.

[2] American Diabetes Association Professional Practice Committee for Diabetes . 2. Diagnosis and classification of diabetes: Standards of care in diabetes-2026. Diabetes Care. (2026) 49:S27–49. doi: 10.2337/dc26-S002 PMC12690183 41358893

[3] Ebrahimi F Kutz A Christ ER Szinnai G . Lifetime risk and health-care burden of diabetic ketoacidosis: A population-based study. Front Endocrinol (Lausanne). (2022) 13:940990. doi: 10.3389/fendo.2022.940990 36093075 PMC9449722

[4] Umpierrez GE Davis GM ElSayed NA Fadini GP Galindo RJ Hirsch IB . Hyperglycaemic crises in adults with diabetes: a consensus report. Diabetologia. (2024) 67:1455–79. doi: 10.1007/s00125-024-06183-8 38907161 PMC11343900

[5] Mendez Y Surani S Varon J . Diabetic ketoacidosis: Treatment in the intensive care unit or general medical/surgical ward? World J Diabetes. (2017) 8:40–4. doi: 10.4239/wjd.v8.i2.40 28265341 PMC5320747

[6] Khan AA Ata F Iqbal P Bashir M Kartha A . Clinical and biochemical predictors of intensive care unit admission among patients with diabetic ketoacidosis. World J Diabetes. (2023) 14:271–8. doi: 10.4239/wjd.v14.i3.271 37035234 PMC10075029

[7] Orban JC Maiziere EM Ghaddab A Van Obberghen E Ichai C . Incidence and characteristics of acute kidney injury in severe diabetic ketoacidosis. PloS One. (2014) 9:e110925. doi: 10.1371/journal.pone.0110925 25338064 PMC4206473

[8] Hua Y Ding N Jing H Xie Y Wu H Wu Y . Association between SOFA score and risk of acute kidney injury in patients with diabetic ketoacidosis: an analysis of the MIMIC-IV database. Front Endocrinol (Lausanne). (2024) 15:1462330. doi: 10.3389/fendo.2024.1462330 39764255 PMC11700799

[9] Chen J Zeng H Ouyang X Zhu M Huang Q Yu W . The incidence, risk factors, and long-term outcomes of acute kidney injury in hospitalized diabetic ketoacidosis patients. BMC Nephrol. (2020) 21:48. doi: 10.1186/s12882-020-1709-z 32050921 PMC7017527

[10] Tian B Chen C Cheng J Wang J Mo J Zhong G . Predictors, morbidity, and healthcare costs of acute kidney injury in diabetic ketoacidosis: A decade-long retrospective cohort study of 464,057 US hospitalizations. Shock. (2026) 65:181–7. doi: 10.1097/shk.0000000000002630 40550726

[11] Huang JX Casper TC Pitts C Myers S Loomba L Ramesh J . Association of acute kidney injury during diabetic ketoacidosis with risk of microalbuminuria in children with type 1 diabetes. JAMA Pediatr. (2022) 176:169–75. doi: 10.1001/jamapediatrics.2021.5038 34842908 PMC8630664

[12] Hursh BE Ronsley R Islam N Mammen C Panagiotopoulos C . Acute kidney injury in children with type 1 diabetes hospitalized for diabetic ketoacidosis. JAMA Pediatr. (2017) 171:e170020. doi: 10.1001/jamapediatrics.2017.0020 28288246

[13] Gameiro J Fonseca JA Outerelo C Lopes JA . Acute kidney injury: From diagnosis to prevention and treatment strategies. J Clin Med. (2020) 9(3):1704. doi: 10.3390/jcm9061704 32498340 PMC7357116

[14] Williams TA Ho KM Dobb GJ Finn JC Knuiman M Webb SA . Effect of length of stay in intensive care unit on hospital and long-term mortality of critically ill adult patients. Br J Anaesth. (2010) 104:459–64. doi: 10.1093/bja/aeq025 20185517

[15] Blanchard F Charbit J Van der Meersch G Popoff B Picod A Cohen R . Early sepsis markers in patients admitted to intensive care unit with moderate-to-severe diabetic ketoacidosis. Ann Intensive Care. (2020) 10:58. doi: 10.1186/s13613-020-00676-6 32430795 PMC7237630

[16] Almohareb SN Aljammaz NA Yousif N Sunbul M Alsemary R Alkhathran L . The effect of renal function on the clinical outcomes and management of patients hospitalized with hyperglycemic crises. Front Endocrinol (Lausanne). (2024) 15:1445040. doi: 10.3389/fendo.2024.1445040 39850482 PMC11754045

[17] Kitabchi AE Umpierrez GE Miles JM Fisher JN . Hyperglycemic crises in adult patients with diabetes. Diabetes Care. (2009) 32:1335–43. doi: 10.2337/dc09-1563 PMC2699725 19564476

[18] Alotaibi A Aldoukhi A Albdah B Alonazi JA Alseraya AS Alrasheed N . Diabetic ketoacidosis treatment outcome and associated factors among adult patients admitted to the emergency department and medical wards at King Abdulaziz Medical City, Riyadh, Saudi Arabia. Cureus. (2020) 12:e10067. doi: 10.7759/cureus.10067 32999787 PMC7522050

[19] Vincent JL Moreno R Takala J Willatts S De Mendonca A Bruining H . The SOFA (Sepsis-related Organ Failure Assessment) score to describe organ dysfunction/failure. On behalf of the Working Group on Sepsis-Related Problems of the European Society of Intensive Care Medicine. Intensive Care Med. (1996) 22:707–10. doi: 10.1007/BF01709751 8844239

[20] Knaus WA Draper EA Wagner DP Zimmerman JE . APACHE II: a severity of disease classification system. Crit Care Med. (1985) 13:818–29. doi: 10.1097/00003246-198304000-00016 3928249

[21] Peduzzi P Concato J Kemper E Holford TR Feinstein AR . A simulation study of the number of events per variable in logistic regression analysis. J Clin Epidemiol. (1996) 49:1373–9. doi: 10.1016/s0895-4356(96)00236-3 8970487

[22] Rahim MA Ananna MA Samad T Zaman S Rouf R Ahmed AU . Acute kidney injury among adult patients with diabetic ketoacidosis in a referral hospital of Bangladesh. BIRDEM Med J. (2018) 8:26–9. doi: 10.3329/birdem.v8i1.35035 40208441

[23] Soltysiak J Krzysko-Pieczka I Gertig-Kolasa A Mularz E Skowronska B Ostalska-Nowicka D . Acute kidney injury and diabetic kidney disease in children with acute complications of diabetes. Pediatr Nephrol. (2022) 38:1643–52. doi: 10.1007/s00467-022-05735-7 36227434 PMC10060302

[24] Fayfman M Pasquel FJ Umpierrez GE . Management of hyperglycemic crises: Diabetic ketoacidosis and hyperglycemic hyperosmolar state. Med Clin North Am. (2017) 101:587–606. doi: 10.1016/j.mcna.2016.12.011 28372715 PMC6535398

[25] Vijayan A Abdel-Rahman EM Liu KD Goldstein SL Agarwal A Okusa MD . Recovery after critical illness and acute kidney injury. Clin J Am Soc Nephrol. (2021) 16:1601–9. doi: 10.2215/cjn.19601220 34462285 PMC8499012

[26] Sabaz MSAS . Identification of factors associated with prolonged stay in the intensive care unit. Med J Bakirkoy. (2021) 17:233–42. doi: 10.4274/bmj.galenos.2021.96658

[27] Chuang CL . Fluid management in acute kidney injury. Contrib Nephrol. (2016) 187:84–93. doi: 10.1159/000442368 26882226

[28] Ahmad R Narwaria M Singh A Kumar S Haque M . Detecting diabetic ketoacidosis with infection: Combating a life-threatening emergency with practical diagnostic tools. Diagnostics (Basel). (2023) 13(14):2441. doi: 10.3390/diagnostics13142441 37510185 PMC10378387

[29] Takahashi K Uenishi N Sanui M Uchino S Yonezawa N Takei T . Epidemiology, microbiology, and diagnosis of infection in diabetic ketoacidosis and hyperosmolar hyperglycemic syndrome: A multicenter retrospective observational study. Diabetes Res Clin Pract. (2024) 212:111713. doi: 10.1016/j.diabres.2024.111713 38772502

[30] Hatton GE Harvin JA Wade CE Kao LS . Importance of duration of acute kidney injury after severe trauma: a cohort study. Trauma Surg Acute Care Open. (2021) 6:e000689. doi: 10.1136/tsaco-2021-000689 34124376 PMC8162072

[31] Mondal T . Diabetic ketoacidosis and its association with comorbid conditions: a review of clinical evidence. J Clin Diabetes. (2024) 8:241. doi: 10.4172/jcds.1000241

[32] Barnett AG Page K Campbell M Martin E Rashleigh-Rolls R Halton K . The increased risks of death and extra lengths of hospital and ICU stay from hospital-acquired bloodstream infections: a case-control study. BMJ Open. (2013) 3:e003587. doi: 10.1136/bmjopen-2013-003587 24176795 PMC3816236

[33] Dasgupta S Das S Chawan NS Hazra A . Nosocomial infections in the intensive care unit: Incidence, risk factors, outcome and associated pathogens in a public tertiary teaching hospital of Eastern India. Indian J Crit Care Med. (2015) 19:14–20. doi: 10.4103/0972-5229.148633 25624645 PMC4296405

[34] Waite MD Fuhrman SA Badawi O Zuckerman IH Franey CS . Intensive care unit-acquired hypernatremia is an independent predictor of increased mortality and length of stay. J Crit Care. (2013) 28:405–12. doi: 10.1016/j.jcrc.2012.11.013 23369520

[35] Yun G Baek SH Kim S . Evaluation and management of hypernatremia in adults: clinical perspectives. Korean J Intern Med. (2023) 38:290–302. doi: 10.3904/kjim.2022.346 36578134 PMC10175862

[36] Mustafa OG Haq M Dashora U Castro E Dhatariya KK Joint British Diabetes Societies for Inpatient Care Group . Management of hyperosmolar hyperglycaemic state (HHS) in adults: An updated guideline from the Joint British Diabetes Societies (JBDS) for Inpatient Care Group. Diabetes Med. (2023) 40:e15005. doi: 10.1111/dme.15005 36370077 PMC10107355

[37] Zhang Y Li J Deng S Zhang Y Guo M Jiang F . Real-world insights from acute management of potassium disorders in diabetic ketoacidosis. Front Endocrinol (Lausanne). (2025) 16:1669400. doi: 10.3389/fendo.2025.1669400 41255534 PMC12620269

[38] Mumtaz H Ejaz MK Tayyab M Vohra LI Sapkota S Hasan M . APACHE scoring as an indicator of mortality rate in ICU patients: a cohort study. Ann Med Surg (Lond). (2023) 85:416–21. doi: 10.1097/ms9.0000000000000264 37008173 PMC10060092

[39] Venkatesh B Pilcher D Prins J Bellomo R Morgan TJ Bailey M . Incidence and outcome of adults with diabetic ketoacidosis admitted to ICUs in Australia and New Zealand. Crit Care. (2015) 19:451. doi: 10.1186/s13054-015-1171-7 26715333 PMC4699354

[40] Monk M Torres J Vickery K Jayaraman G Sarva ST Kesavan R . A comparison of ICU mortality scoring systems applied to COVID-19. Cureus. (2023) 15:e35423. doi: 10.7759/cureus.35423 36987484 PMC10040236

[41] Benoit SR Zhang Y Geiss LS Gregg EW Albright A . Trends in diabetic ketoacidosis hospitalizations and in-hospital mortality - United States, 2000-2014. MMWR Morb Mortal Wkly Rep. (2018) 67:362–5. doi: 10.15585/mmwr.mm6712a3 29596400 PMC5877353

[42] Umpierrez G Korytkowski M . Diabetic emergencies - ketoacidosis, hyperglycaemic hyperosmolar state and hypoglycaemia. Nat Rev Endocrinol. (2016) 12:222–32. doi: 10.1038/nrendo.2016.15 26893262

[43] Esposito P Cappadona F Prenna S Marengo M Fiorentino M Fabbrini P . Acute kidney injury in hospitalized patients with real-life analysis of incidence and clinical impact in Italian hospitals (the SIN-AKI study). Sci Rep. (2025) 15:14261. doi: 10.1038/s41598-025-96236-8 40274969 PMC12022043

[44] Schulman IH Chan K Der JS Wilkins KJ Corns HL Sayer B . Readmission and mortality after hospitalization with acute kidney injury. Am J Kidney Dis. (2023) 82:63–74.e1. doi: 10.1053/j.ajkd.2022.12.008 37115159 PMC10293057

[45] Malhotra R Kashani KB Macedo E Kim J Bouchard J Wynn S . A risk prediction score for acute kidney injury in the intensive care unit. Nephrol Dial Transplant. (2017) 32:814–22. doi: 10.1093/ndt/gfx026 28402551

[46] Kidney Disease: Improving Global Outcomes (KDIGO) Acute Kidney Injury Work Group . KDIGO clinical practice guideline for acute kidney injury. Kidney Int Suppl. (2012) 2:1–38.

[47] Adiyeke E Ren Y Ruppert MM Shickel B Kane-Gill SL Murugan R . A deep learning-based dynamic model for predicting acute kidney injury risk severity in postoperative patients. Surgery. (2023) 174:709–14. doi: 10.1016/j.surg.2023.05.003 37316372 PMC10683578

[48] Austin PC Steyerberg EW . Events per variable (EPV) and the relative performance of different strategies for estimating the out-of-sample validity of logistic regression models. Stat Methods Med Res. (2017) 26:796–808. doi: 10.1177/0962280214558972 25411322 PMC5394463

